# Editorial board interlocking across the social sciences: Modelling the geographic, gender, and institutional representation within and between six academic fields

**DOI:** 10.1371/journal.pone.0273552

**Published:** 2022-09-02

**Authors:** Manuel Goyanes, Luis de-Marcos, Márton Demeter, Tamás Toth, Beatriz Jordá

**Affiliations:** 1 Departamento de Comunicación, Universidad Carlos III, Madrid, Spain; 2 Departamento de Ciencias de la Computación, Universidad de Alcalá, Alcala de Henares, Spain; 3 University of Public Service, Budapest, Hungary; University of Siena, Italy, ITALY

## Abstract

Editorial boards play a key role in the production, dissemination, and promotion of scientific knowledge. The cross-presence of scholars in different journals, known as editorial board interlocking, maps the connections between such bodies of governance. Former research on this topic is typically restricted to individual disciplines and has failed to consider the relevance of potential interlocking between related, but different academic fields. Further, although existing studies note a significant lack of diversity in editorial board representation, they mainly focus on a single dimension, such as gender or geography. This study addressed these knowledge gaps by offering a complex cross-disciplinary approach to the geographical, gender, and institutional compositions of editorial boards, with a specific emphasis on within- and between-fields editorial board interlocking. We used graph and social network analysis to examine editorial board connections between 281 top journals (13,084 members and 17,092 connections) of six disciplines: communication, psychology, political science, sociology, economics, and management. We found substantial differences in terms of field connections, ranging from sociology with 42% interlocking with other fields, to management with only 11%. Psychology is significantly less connected to the other five disciplines. The results also show a clear overrepresentation of American institutions and native English-speaking countries in all fields, with Harvard, Columbia, Cornell, Stanford, UC Berkeley, and New York University forming a well-connected central cluster. Although female scholars are underrepresented, there are no significant differences in terms of positioning in the network. Female scholars are even employed in more central positions than male scholars in psychology, sociology, and management. Our findings extend the literature on editorial board diversity by evidencing a significant imbalance in their gender, geographical, institutional representation, and interlocking editorship both within and between fields.

## Introduction

The growing importance of publication output in leading peer-reviewed journals [1], and the increasing demand for research diversity in academia [2], have spurred scholarly interest in editorial board representation [1]. Research into the roles and functions of editorial boards has suggested that these bodies of governance play a decisive role during the peer-review process [3], serving as gatekeepers of knowledge [4], and ultimately setting a journal’s research and thematic direction [5].

Although editorial board (EB) members are typically recruited according to their “measured” academic achievements [6], research has empirically shown that their appointment is increasingly concentrated in a handful of Western institutions and academic profiles [7]. This systematic cross presence of editorial board members in different journals is academically known as editorial board interlocking (EBI) [8], and represents, according to a number of studies, a growing threat to the diversity of research, as it is theoretically assumed that the homogeneous power structure of these bodies may jeopardize the pluralism of scholarly publishing [5], thus widening the standardization of research paradigms within fields, and, ultimately, guillotining scientific progress [9].

Thus far, the growing literature on editorial board interlocking has empirically examined interlocking editorships *within* several research fields of the social sciences [10]. This strand of research has not yet explored the connections both *within* and *between* academic fields, nor the scientific structure and institutions of the dominant scholars, thus remaining unclear the power structure that governs major disciplines in social sciences. This study addresses this gap in the literature and examines editorial interlocks in the leading journals of six major fields.

Specifically, this study focuses on disciplines that are frequently analyzed in terms of editorial board composition but lack cross-discipline analyses: communication [2, 9, 11–13], psychology [14–16], political science [17, 18], sociology [19, 20], economics [8, 21, 22], and management [1, 4, 5]. More specifically, we employ social network analysis with the aim of (1) mapping the EBI of the above six fields; (2) identifying the representation of EB members in terms of gender, affiliation, and nationality; and (3) finding the connections between fields and their magnitude.

Our empirical results show that a typical central EB member is a male scholar from an elite American university who contributes to many different journals. Women are less likely to be editorial board members across all the analyzed disciplines, and influential affiliations are different for each field, with no central actor but rather a set of strong connections between relevant institutions. Overall, our results indicate a relatively loose but still existing network of notable scholars and elite institutions who occupy gatekeeper positions across analyzed fields. We argue that the uneven distribution of gatekeeping and a considerable interlocking across disciplines might hinder the balanced representation of gender and regions, and, ultimately, it might decrease the diversity of perspectives in the social sciences.

## Literature review

### Editorial boards: Role, function, and diversity

Academic journals are typically considered the main spaces for the dissemination of scientific knowledge [23]. Research publications are the major outcome of scientific progress in today’s academia, and publishing manuscripts in top-tier journals has become a direct mechanism for promotion and hiring decisions [1]. Against this backdrop, a growing number of scholars have started to investigate the power structures of academic journals to shed light on the processes of research domination. Accordingly, scholars have focused on the study of editorial boards, the gatekeepers of knowledge [4] and the bodies of governance that set the research and thematic direction of scientific journals [3, 5].

Extant research has shown that positions on the EBs of top-tier journals are typically held by prominent scholars with great expertise and experience [1]. Rational motivations for their recruitment include “measured” academic achievements, such as high academic performance [6, 21]. However, critics have long observed that such appointments are far from open, and that other factors such as research bounds, connections, and scientific similarity, may be at play. Accordingly, it has been suggested that a lack of diversity of editorial board members may jeopardize research pluralism: EBs may make biased intergroup evaluations stemming from the academic backgrounds and perspectives of their own members [1]. The genuine internationalization and diversity of science, two of the key components of knowledge creation and scientific progress, may thus be at risk [e.g., 2, 9].

Several studies have emphasized the low gender and institutional diversity of editors [24]. For instance, male scholars from Global North, predominantly US-based, institutions dominate the EBs of top-tier journals [e.g., 7, 13, 25], while researchers from the periphery and female academics have a moderate representation, in terms of both board membership and authorship. This Western hegemony in EBs replicates that of knowledge production, which is also dominated by Anglo-American universities [26, 27]. Clauset et al. [28] point out that a few prestigious institutions have placed a high number of faculty members in some disciplines, and their increased institutional prestige subsequently leads to increased faculty production. All in all, not only do Anglo-Saxon institutions monitor the refereeing process of top-tier journals, but also produce the lion’s share of global publication output [2], despite the growing numbers of China. The share of Chinese scientific output is significantly lower in the social sciences, however, than in the hard sciences, and their share of the top cited papers still lags behind the US or Europe [29].

Studies have demonstrated that there is less female representation on EBs for high impact journals, and that it tends to decrease the higher the position [5]. Metz et al. [4] found that women only held 19% of the EB positions in their sample of 52 management journals. Altman and Cohen [24] suggest the dominance of EBs by male scholars in 6,090 journals from seventeen publishers, as well as the dominant positions of the US and Great Britain. In terms of authorship, studies show that female scholars are especially underrepresented in disciplines related to power and policy, and that they comprise a higher share in fields focused on social care [30, 31]. From the perspective of gender equality, the underrepresentation of females on EBs may deprive academia of the contributions and perspectives of female scholars [e.g., 32].

### Editorial board interlocking and why it matters

The crossed presence of scholars on more than one EB is a phenomenon referred to as editorial board interlocking [8]. EBI is considered the main indicator of inter-journal network ties [22], and has increasingly been used to showcase the network structures of EBs, and their patterns of power concentration. When the same scholar is a board member of various journals, they become part of “an elite within an elite” of highly influential academics [1, p. 635]. The high-level criteria by which these prestigious board members manage or even revise manuscripts may improve the quality of scientific work [10, 33]. In many cases, editorial board members are invited to review a given number of papers annually prior to their appointment, and even if they do not conduct the reviews themselves, social identity theory [34], suggests that they might invite reviewers from their own academic circles.

Moreover, research has suggested that some individuals may also be invited to join editorial boards based on their international excellence and prestige [2], typically handling reviews, but not reviewing. In this case, inviting top-notch scholars is believed to improve a journal’s prestige and to define the area of research in which the journal aims to excel. While “normal” editorial board members improve journal quality either by reviewing manuscripts or managing the review process, academic stars [12], typically boost a journal’s excellence and prestige. On this backdrop, many studies point out that EBI, together with the low diversity of EBs described above, may encourage the emergence of “invisible colleges” [35], which may subconsciously favor specific types of submissions and develop similar paradigms and paths of evolution in interlocked journals, impoverishing research diversity.

Studies have suggested that EBI has a greater influence over research production for the social sciences and humanities than for hard-science disciplines [8]. Research on EBI has thus primarily focused on examining this phenomenon in the social sciences, finding significant interlocks in disciplines such as economics [8], finance [21], knowledge management [10], or communication [7]. Recent studies [36, 37] have also compared networks of editors and authors, and co-citations of different disciplines, namely, economics, and information and library sciences, as to show how they are associated. These studies demonstrate that co-citation networks, interlocked authors, and especially editors, are associated.

In summary, research into scientometrics has highlighted the low diversity of EBs and the connections between journals and editors through EBI [e.g., 4, 8]. While these studies have offered insightful evidence about how interlocks may potentially create invisible colleges, they have also been predominantly focused on connections within fields. The literature has not, to this point, investigated the connections between fields through EBI, nor the characteristics and institutions of the dominant scholars. Our study analyzes six disciplines in order to fill this gap. Specifically, this study focuses on disciplines that are frequently analyzed in terms of editorial board composition but lack cross-discipline analyses. Accordingly, this study focuses on six highly analyzed social science fields: communication [2, 9, 11, 13], psychology [14–16], political science [17, 18], sociology [19, 20], economics [8, 21, 22], and management [1, 4, 5]. Accordingly, we pose the following research questions:

RQ1: What is the geographic and gender representation of the editorial boards across the six fields?
RQ2: What institutions are in central positions in the social networks of the six fields?
RQ3: What are the connections a) within, and b) between fields through EBI?

## Methods

### Data collection & sampling

The data used in this study comes from the public webpages of the selected journals. We used the Journal Citation Report (JCR) as a source for journals because it has the most influential ranking. We used 2019 for the analysis because it was the most recent data available when we started our research. Journals indexed by the JCR are grouped into categories. We selected six categories of the JCR as the fields examined for this study: communication, psychology, political science, sociology, economics, and management. The fields were selected on the basis of their academic proximity, tradition within the literature of editorial board interlocking, and their reach within the social sciences.

Since journals can be listed in more than one category, the field for each journal was determined by selecting the first category listed by the JCR SSCI which was among the fields considered for this study. For instance, if a selected journal was listed under engineering and management, we labeled it as management because engineering is not part of the study. Categories were defined by Clarivate Analytics (formerly known as ISI) on the basis of several indicators, such as the title of the journal and citation patterns [38, 39]. The order of categories was defined by relevancy; thus, if a journal was listed under both communication and political science (in this order), we coded it under communication as the more relevant category. Nineteen journals (6.76% of the sample) were cross listed in two of the six categories of the study.

Journals are divided into four different quartiles by their impact factor for each category of the JCR. In 2019, the population of the six categories under study included 1,100 JCR-ranked journals: 92 journals for communication, 78 for psychology, 181 for political science, 150 for sociology, 373 for economics, and 226 for management. In order to make such large number of journals and the data collection feasible, we selected a representative, stratified random sample of the 1,100 indexed journals, with a margin of error of 5%, taking 25% of journals evenly distributed between quartiles, so that journals with different degrees of influence were represented in the sample. Accordingly, the journals sample is representative of the journal population. This random selection was implemented by using a random number generator. In total, our proportional random sample consisted of 281 journals of the 1,100: 25 for communication, 20 for psychology, 44 for political science, 36 for sociology, 99 for economics, and 57 for management. S1 Appendix presents the journals included in this study. For each journal in the sample, we coded all the members of its EB and built an adjacency matrix of connections between EB members and journals.

It was not reasonably feasible for this study to gather and analyze the data from the complete network of EBs, so sampling was used. The sampling goals for this study were (1) to obtain a representative network that reflects both structural network properties and community structure, and (2) to preserve the distribution of node attributes. Biases in sampling may affect results in different ways, however, and there is no perfect method that accurately balances network structure, communities, and attribute distribution [40]. Several methods also require access to, or the capacity to explore, the complete network, which is not always possible.

Sampling methods for graphs include random selection and crawling. Random selection chooses nodes or edges using either uniform, probability (e.g. degree-based) or hybrid distributions. Crawling methods sample the graph by exploring it. Among them, breadth sampling explores all the edges of current nodes, depth sampling prioritizes the first edge of current nodes, and random walks explore edges uniformly at random. Forest Fire Sampling is a probabilistic version of breadth sampling that explores edges with a given probability. Evidence shows that node sampling is better at preserving degree distributions [41]. Edge sampling is better at preserving the communities of connected components, although it performs poorly when preserving other structural properties. No method preserves connected components particularly well. Uniform node samples result in sparsely connected networks, while crawled samples lead to over-sampling of high-degree nodes. Advanced sampling methods such as Metropolis-Hastings random walk [42] compensates for crawling bias, while stratified samples [43] better preserve community structure. Unfortunately, they may not be feasible because they require access to the complete graph or expedited crawling. When it comes to attribute representation, research suggests that differences in group size and homophily (the tendency of nodes to form connections with others that share the same values) in the original network affect the representativity of attributes and guide the best sampling method to use [44]. In heterophilic networks with unbalanced groups, which we can assume is the case for the social network of EBs, random walk and edge sampling perform similarly well.

Given the nature of the bipartite network of EBs, it is possible to sample and explore journals if we use a source for journals such as the JCR and then identify the EBs as reported on the journal’s web page. However, it is difficult to sample and explore scholars, since sources of researchers are not as common or widely accepted, and it would be necessary to obtain the journals in which individual scholars serve as EB members in order to explore them all. This may be reported on personal or institutional web pages, requiring specific and unreliable searches. Random sampling of edges was thus not possible for this study.

As we can then see, different sampling strategies work for certain goals better than others. We tried to mitigate the limitations of data acquisition and the limitations built-in for each sampling method, while reasonably preserving structural properties and attribute distributions. We thus combined random uniform node sampling and breadth-first sampling. The sampling method used was an adaptation of the Random Node Neighbor (RNN) for a bipartite network. We selected a random set of journals (random-first sampling) and sampled its out-going edges (breadth sampling). RNN keeps the structural properties of the sampled graph for a sample size over 15% [41]. Although RNN is appropriate for this research, it may not fully reflect community structure and attribute distribution, and as such we acknowledge it as a limitation. We must be careful when interpreting results in any study using graph sampling, since social network analysis usually requires complete graphs, and any sampling may eventually miss important individual nodes or include bias when capturing network properties.

The final sample included 15,084 scholars and 17,092 memberships. Table 1 summarizes the features of the sample for each field and for the overall network. The overall network includes all sampled journals, members, and memberships. We coded the affiliation name and affiliation country for EB members, as reported in the public webpage of the journal. The institution was not reported for 91 scholars and was coded as missing for the data analysis. Similarly, country was not reported for 35 entries. We also coded the gender of EB members using a gender API (https://gender-api.com/). The API returns a prediction of gender based on the name and an estimation of accuracy, which is a measure between 0 and 100 of the reliability based on the API internal database. We coded the gender when the accuracy returned was greater than 75%. Since API prediction is mostly based on the first name, we did not use the API for EB members who were reported in journals using only initials. In total, 14669 entries were “genderized” using the API and the gender of 415 EB members remained undetermined.

**Table 1:** Sample for each field and for the complete network.

	Comm.	Psych.	Pol. Sc.	Soc.	Eco.	Man.	All
EB members	1586	938	1650	1459	3898	5750	15,084
Journals	25	20	44	36	99	57	281
Edges	1836	957	1762	1540	4267	6730	17,092
Institutions	617	425	594	552	1075	1512	2850

*Notes*: A scholar can part of the EB of journals in two or more fields. Edges represent an EB membership between a scholar and a journal.

### Social network analysis & metrics

Social network analysis (SNA) investigates the social structure of networks using graph theory. The social network of scholars (EB members) and journals can be represented as a graph in which nodes represent actors (scholars and journals) and edges represent their shared EB memberhips. This network is shown in a bipartite graph, in which the edges only connect the nodes of two different sets (scholars with journals). Bipartite networks are sometimes called affiliation networks or two-mode networks.

SNA provides different metrics with which to quantify the network properties of individual nodes and of complete networks. The individual properties of nodes (EB members and journals) were analyzed using degree, closeness centrality and betweenness centrality, as used in previous studies [8, 10, 21, 45, 46]. Degree is the total number of a node’s connections. In our case, journals degree represents the number of members on their EB. For scholars, the degree is the number of journals in which they act as members of the EB. Since the degree indicates the number of nodes directly connected to a given actor, it then determines the number of EB or journals that can be reached or influenced in a single action. Actors with a very high degree are usually called whales.

Closeness centrality represents the distance to the center of the network for a given node. It can be regarded as a measure of how long it takes to spread information from the node. In this study, closeness centrality is normalized [47]. The maximum possible value is one, showing that the node is connected to all other nodes by the minimum possible distance. In a bipartite network the minimum distance for any node is two to other nodes in the same set, and one to nodes in the opposite set. Higher values then represent a more central position in the network. In our case, higher values of centrality for scholars and journals are indicative of the capacity of actors to exert influence in the network by spreading their ideas and convictions faster to other relevant actors.

Betweenness centrality measures the number of times that a given node is part of the shortest path between two other nodes. It is a measure of the influence of a node over the resources and the flow of information in the network. Betweenness shows which nodes act as a bridge between different parts of the network. For example, a journal or EB member with high betweenness acts as a connector between two parts of the network that may otherwise be unconnected or distant, and plays a relevant role in whether information and ideas spread through the network. The betweenness centrality of a given node is computed as the sum of the fraction of all pairs of the shortest paths that go through the node. In this study, betweenness is normalized by the maximum possible value, which is total number of pairs of nodes [47].

Since this study collected the social network of EB memberships of six different fields, it is possible to analyze the similarities and differences between the fields using the SNA metrics that quantify the structural properties of complete networks. It is also possible to analyze and compare fields with the overall network that includes all journals and all scholars from the six fields. The structural properties of the networks for each field, and for the overall network, were compared using the following network metrics: density, the clustering coefficient, average path length, diameter, number of connected components, average degree, average closeness centrality and average betweenness centrality. Density is the ratio between the number of edges in the graph and all possible edges. It is a measure of the overall number of connections. Clustering measures the intensity of local connections with the closest nodes. In our case, density and the clustering coefficient are indicative of the level of a field’s overall cohesion. Fields with low density can still have higher values for clustering coefficients by having tightly connected subgroups working independently which are only sparsely connected through EBIs.

Average path length is the average of the shortest path between all possible pairs of nodes. It provides an estimation of the distance between participants. Diameter is the distance between the two farthest nodes in the network (i.e. the longest path of all shortest paths). In our case, low values for average path length and the diameter of the network of a given field indicate that information and ideas can spread fast. The combination of all these metrics also provides an estimation of the organic nature of each field. Networks of human activity usually show low density, but also short average path lengths, small diameters, and high clustering coefficients. The networks of EBIs for each field should present these features.

A connected component is a set of nodes linked to each other by paths. The number of connected components is the number of such sets in the network. Components represent areas of activity that are unconnected through EBI, and the number of connected components can then provide an estimation of the level of fragmentation of a given network. In our case, a field with a high number of connected components represents a substantial number of groups working independently that do not share any kind of information, ideas, or interests through EBI. Conversely, fields with only one connected component may present a lower level of diversity since interest may be shared across the whole network.

The corresponding averaged values of the metrics previously defined for all nodes are average degree, average closeness centrality and average betweenness centrality. An analysis of all network metrics is used in this study to determine the similarities and differences in terms of connectivity, cohesion, and distance both between fields and with the overall network.

A projection of a bipartite network is a one-mode network that contains the nodes of one set and the edges between each pair of nodes only if they have a common affiliation in the original bipartite network. A weighted projection includes an integer-valued attribute at each edge to indicate the number of shared affiliations between the connected nodes. For instance, two journals are connected in a projection if they share EB members, and the weight of the edge indicates how many members are common to both journals. EB members and journals have additional properties, such as affiliation, country or field, which can be used to build projections of these relevant features. We used the weighted projections of institutions and fields to find these relevant actors and analyze their connections through EBI.

Since projections of institutions may have a significant number of nodes and edges, we used slicing to find the subsets of institutions which have intense ties. A slice is a subgraph that only has edges with a weight equal to or larger than a given parameter, as well as the nodes that connect all those edges. Slices show the presence of cohesive groups in which connections are more intense [48]. We analyzed the slices of institutions for each field, and for the overall network.

### Analytical tools

This study used SNA to analyze the bipartite network of EB memberships formed by scholars and journals. We used the Python’s NetworkX package (https://networkx.org/) to create and analyze the graphs, compute the network metrics, and create the weighted projections. Graph figures were produced using the Gephi network visualization and exploration software [49]. Visualizations were created using the ForceAtlas 2 layout implemented in Gephi. The visualization of social networks conveys the results of the analysis, showing patterns in connections and properties of actors.

We used the Pearson correlation to measure the linear correlation between variables, and the Kruskal-Wallis nonparametric test for the difference in medians of network metrics for different samples (gender & country). Non-parametric tests comparing medians are adequate because network metrics do not usually follow normal distributions.

## Results

### Social networks and structural properties

**Fig 1:**
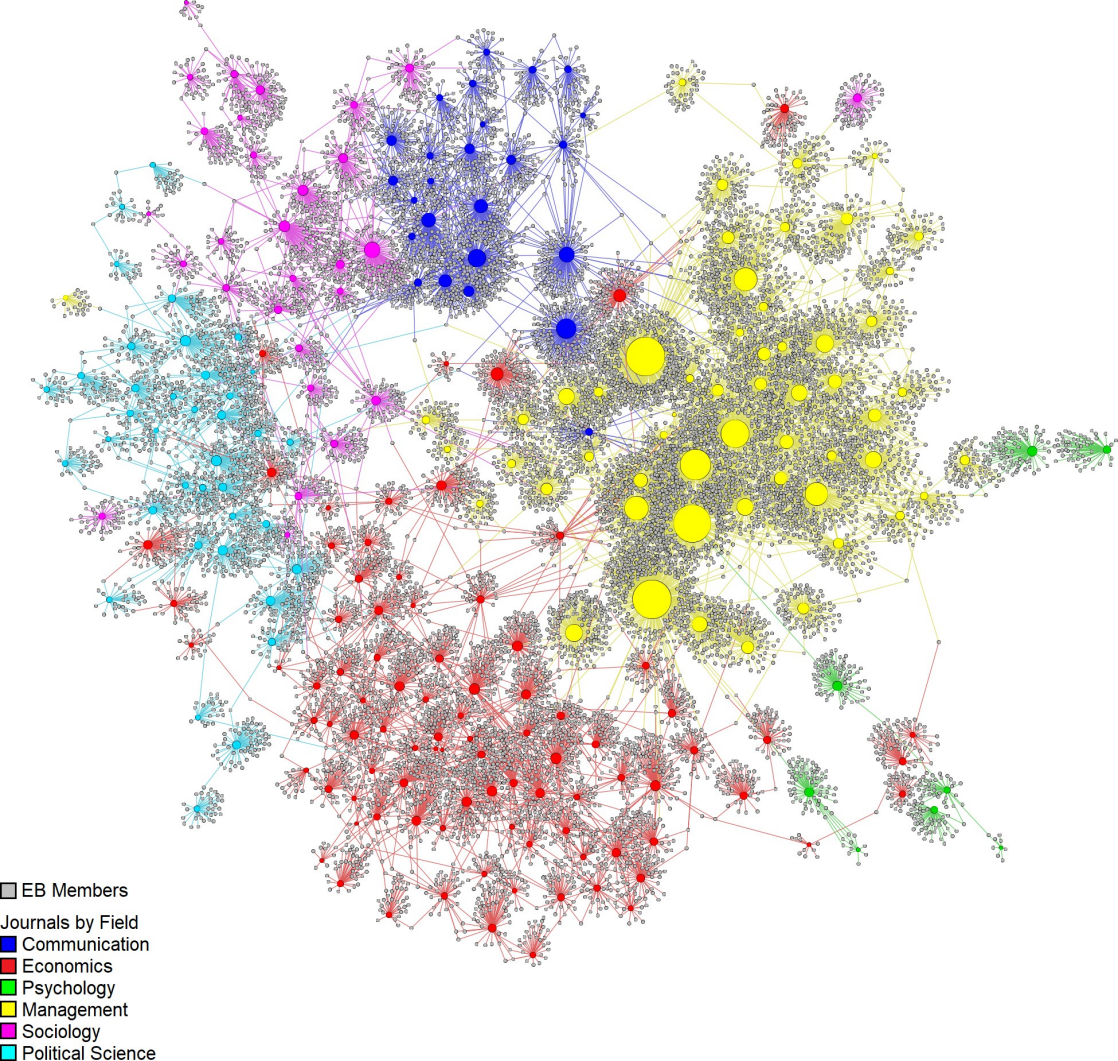
Social network of journals and EB members for the six fields. Only the main connected component is represented.

**Fig 2:**
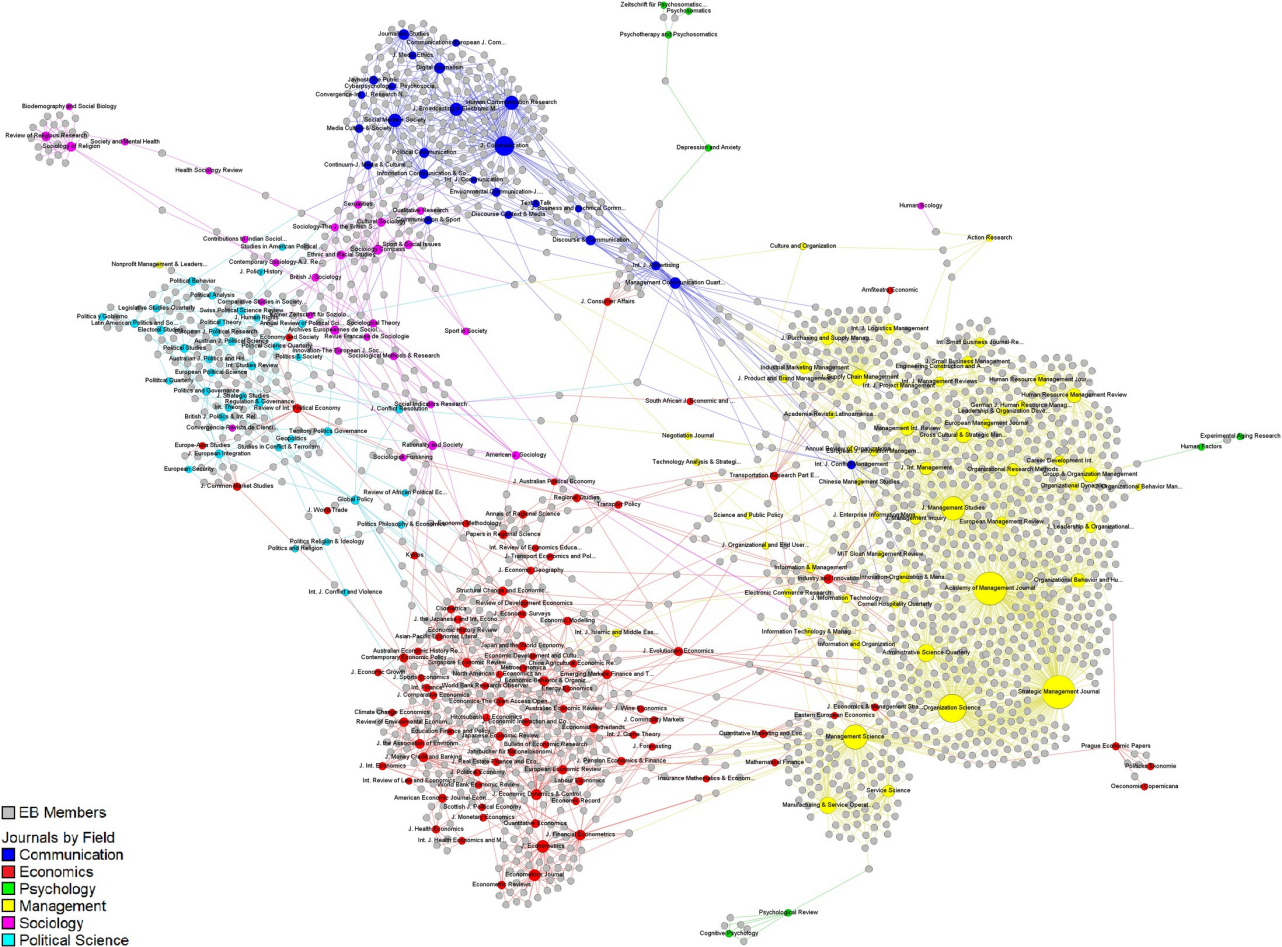
Social network of EBIs for the main connected component.

The final graph presents 15,365 nodes (281 journals, 15,084 EBs) and 17,092 edges forming 31 connected components. The main connected component includes 14,255 nodes (246 journals, 14,009 EBs) and 16,022 edges with 2018 EBIs (1667 scholars interlocking two or more EBs). A second component includes five neuropsychology journals and 236 EBs with 10 interlockings. A third component includes two journals (*Youth & Society*, *and Journal Studies on Alcohol and Drugs*) and 106 EBs with only one interlocking. The remaining 28 components include only one journal each, comprising a total of 733 EBs do not interlock with any other journal. It is difficult to represent the complete graph here because of its size. Fig 1 shows the unlabeled graph of the main connected component. The corresponding complete labeled graph is included as in S1 Fig. (as a high-resolution image and as a vector graphic image). Fig 2 presents the social network of EBIs for the central connected component only.

Visual interpretation suggests that journals tend to cluster by field. Management, economics, communication, and political science form dense clusters. Although sociology journals are also grouped together, they form more connections with communication and political science. Psychology journals are underrepresented in the main connected component with only eight journals, which also occupy positions on the periphery of the network. The central connected component represents the main area of EB activity connected through interlocking. It forms a group that can work together sharing information, ideas, or interests through EBI. The presence of a big central component, like the one observed here, may indicate a lower level of diversity since interests, practices, policies, and beliefs can be shared across the EB of the whole network.

The density of the network for each of the fields is low (Table 2). Economics returns the lowest density (0.011), and psychology is the highest (0.051). The density of the complete network formed by the six fields is the lowest (0.004), while the average clustering coefficient is high (0.829). The clustering coefficient for the networks of all fields is high (range 0.804–0.951). High clustering coefficients reflect the intensity of local connections, suggesting that all nodes tend to be tightly connected to their closest neighbors although the number of overall possible connections (density) is low. In the context of editorial boards, low clustering means that ideas can spread fast, while interests are also strongly shared among journals that are close in the network because they share a substantial number of EB members. Local research communities exist in the overall network and in each field, forming groups of scholars interlocked by common sets of ideas and practices. The presence of a single central cluster also suggests that such communities of local research practice are still connected to the big network, albeit through weaker ties and longer communication paths. Other structural metrics such as short average path length, low density and small diameter are present in the networks of all fields, and also in the complete network (Table 2), suggesting that they can be characterized as a small-world [50–53].

**Table 2:** Metrics of the networks for the six fields and for the complete network.

	Comm.	Psych.	Pol. Sc.	Soc.	Eco.	Man.	All
Density	0.046	0.051	0.024	0.029	0.011	0.021	0.004
Clustering coeff.	0.812	0.951	0.887	0.905	0.862	0.804	0.829
Avg. degree	2.279	1.998	2.080	2.060	2.135	2.318	2.225
Avg. closeness Centrality	0.396	0.437	0.273	0.324	0.258	0.360	0.249
Avg. betweenness Centrality	0.001	0.007	0.002	0.002	0.001	<0.001	<0.001
Avg. path length	5.073	3.733	7.425	6.225	7.850	5.626	8.097
diameter	8	10	16	12	18	12	20
# Connected components	1	11	7	10	13	4	31
EB members in main component	1586	236	1532	1252	3560	5651	14009
Journals in main component	25	5	38	27	87	54	246

### Editorial board interlocking within and between fields

**Fig 3:**
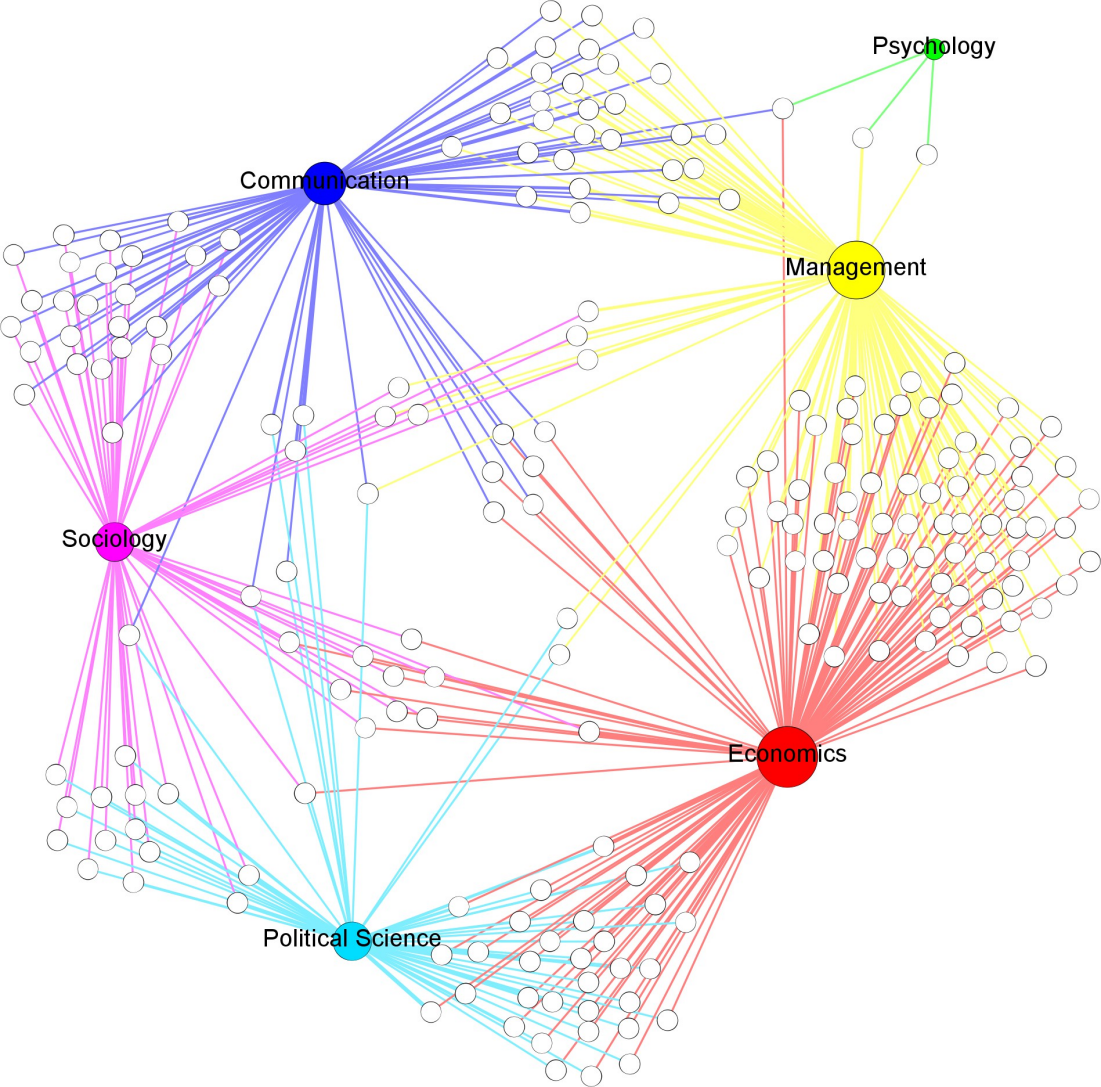
Connections between fields through EBIs. White nodes represent scholars who act as members of EBs in journals of more than one field. Size of field nodes is proportional to degree (number of interdisciplinary scholars).

The network presents 2018 EBIs of 1667 scholars who interlock in two or more journals. 1802 EBIs occur within fields, and 216 EBIs are between fields. Table 3 presents the EBIs within and between all fields. Fig 3 shows the connections between scholars and fields through EBIs. 208 scholars interlock in the EBs of journals from two or more fields (four scholars interlock three fields, and 204 scholars interlock two fields). All fields are connected through EBIs, except for psychology, which only forms connections with sociology and political science in our sample.

**Table 3:** Editorial board interlockings within and between fields.

	Comm.	Psych.	Pol. Sc.	Soc.	Eco.	Man.
Communication	250					
Psychology	1	10				
Political Sc.	7 (1)	0	112			
Sociology	26 (2)	0	16 (1)	81		
Economics	7	1	33 (6)	11 (2)	369	
Management	30 (1)	2	3	6 (1)	73 (5)	980

*Notes*: 2018 EBIs of 1667 scholars. 1802 EBIs within field and 216 EBIs between fields. 204 scholars interlock two fields and 4 scholars interlock three fields. The second number in each cell, when present, represents the number of journals listed in both categories.

**Fig 4:**
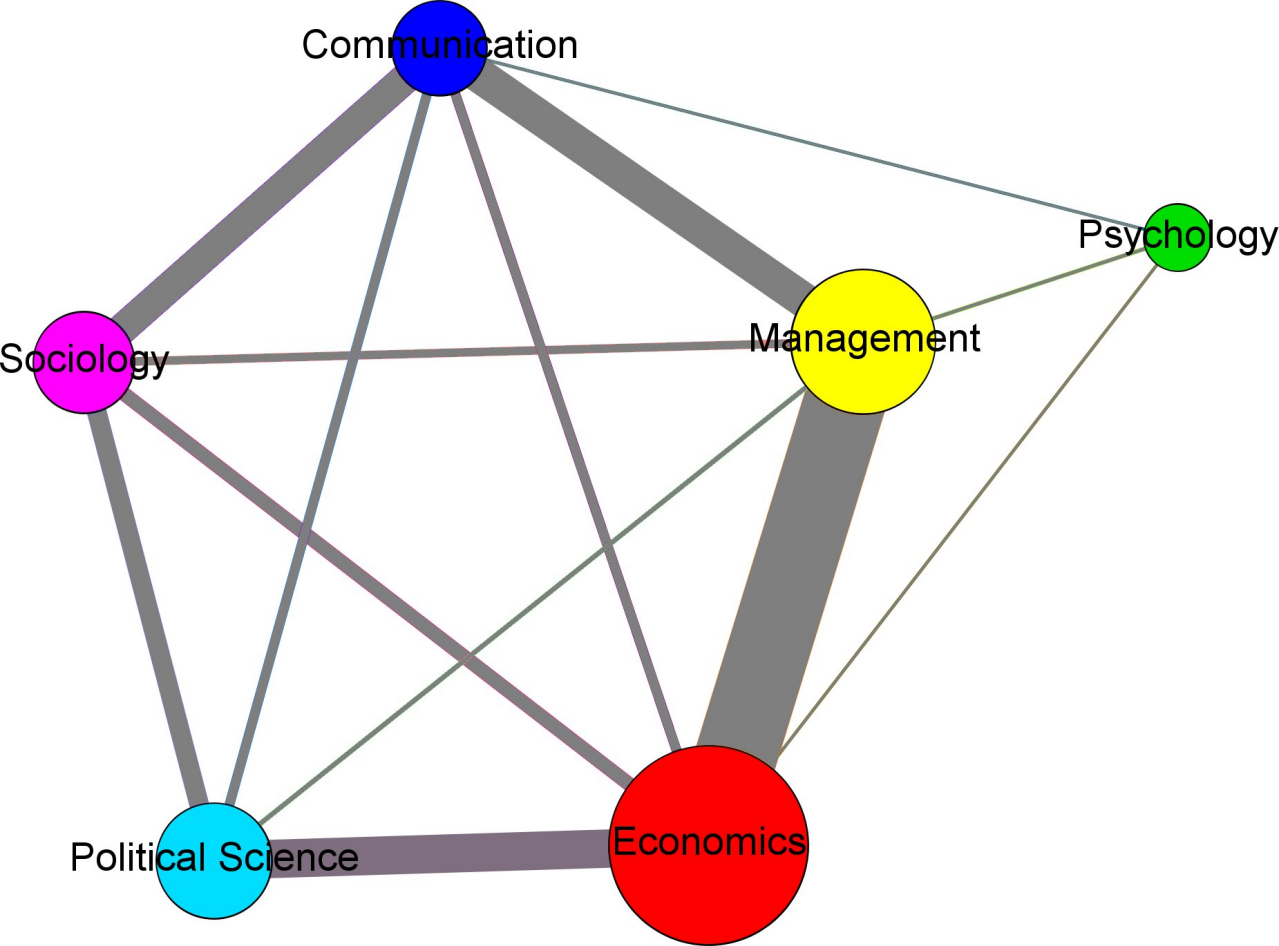
Connections between fields. Size of fields is proportional to the number of journals in the main component. Thickness of connections is proportional to the number of shared EBs.

The network projection of fields through their EBI (Fig 4) has a density of 0.867. Assuming that the number of EBIs between fields determines the intensity of communication between them, connections are stronger at the edges of Fig 4 (e.g., communication has more interlockings with sociology and management). In terms of interdisciplinarity, sociology forms more connections with other fields (42.14% between fields vs 57.86% within), followed by political science (34.50% between vs 65.50% within), psychology (28.57% between vs 71.43% within), economics (25.30% between vs 74.70% within), communication (22.12% between vs 77.88% within), and management (10.42% between vs 89.58% within). The differences between disciplines in terms of inter- and intra- connections are then substantial. Connections between the fields may be partially explained by journals listed in two categories of the study in the JCR (Table 3). However, the limited presence of cross-listed journals in the sample (only 19) precludes statistical analyses, and interpretation should be based on observation. For example, the number of cross-listed journals in political science, sociology and management is similar while the latter field presents a substantially smaller number of EBI with journals of other fields, suggesting that journals listed in two categories do not necessary interlock with journals in the secondary category.

### Gender and geographic distribution

There are fewer female editorial board members in the six fields as well as in the overall sample (Table 4). Sociology and communication are the most balanced fields with 38% female scholars as members of EBs. Economics has the most unbalanced gender distribution, with only 20% of female scholars. When it comes to network metrics, Kruskal-Wallis non-parametric tests show that there is no difference for degree (H = 1.00, p = 0.318) or betweenness centrality (H = 1.04, p = 0.308). The results for the overall network regarding closeness centrality (Table 5) suggest that male scholars are closer to the center. This contrasts with the differences in each field, with female scholars having a higher closeness centrality in psychology, sociology and management. In economics, male scholars present a higher closeness centrality than female scholars. There are no statistical differences in communication and political science. Although the differences in psychology and sociology are larger, the networks for their fields are smaller when compared with management and economics, and such differences are outweighed in the overall network.

**Table 4:** Gender representation of EBs for each field and for the complete sample.

	Comm.	Psych.	Pol. Sc.	Soc.	Eco.	Man.	All
Female	604	270	521	562	758	1401	4064
Male	975	552	1102	891	2930	4299	10,605
Unknowns	7	116	27	6	210	50	415

*Notes*: A scholar can be on the EB of journals in two or more fields

**Table 5:** Differences in gender for closeness centrality for each field and for the overall network.

	Median female	Median Male	H
Communication	0.391	0.392	0.79
Psychology	0.475	0.449	4.04(*)
Political Sc.	0.280	0.278	0.138
Sociology	0.319	0.310	14.69(**)
Economics	0.257	0.270	50.59(**)
Management	0.366	0.359	10.65(**)
Overall	0.244	0.248	39.69(**)

*Notes*: Results of Kruskal-Wallis H test.

(*) p< 0.05,

(**) p< 0.01

**Fig 5:**
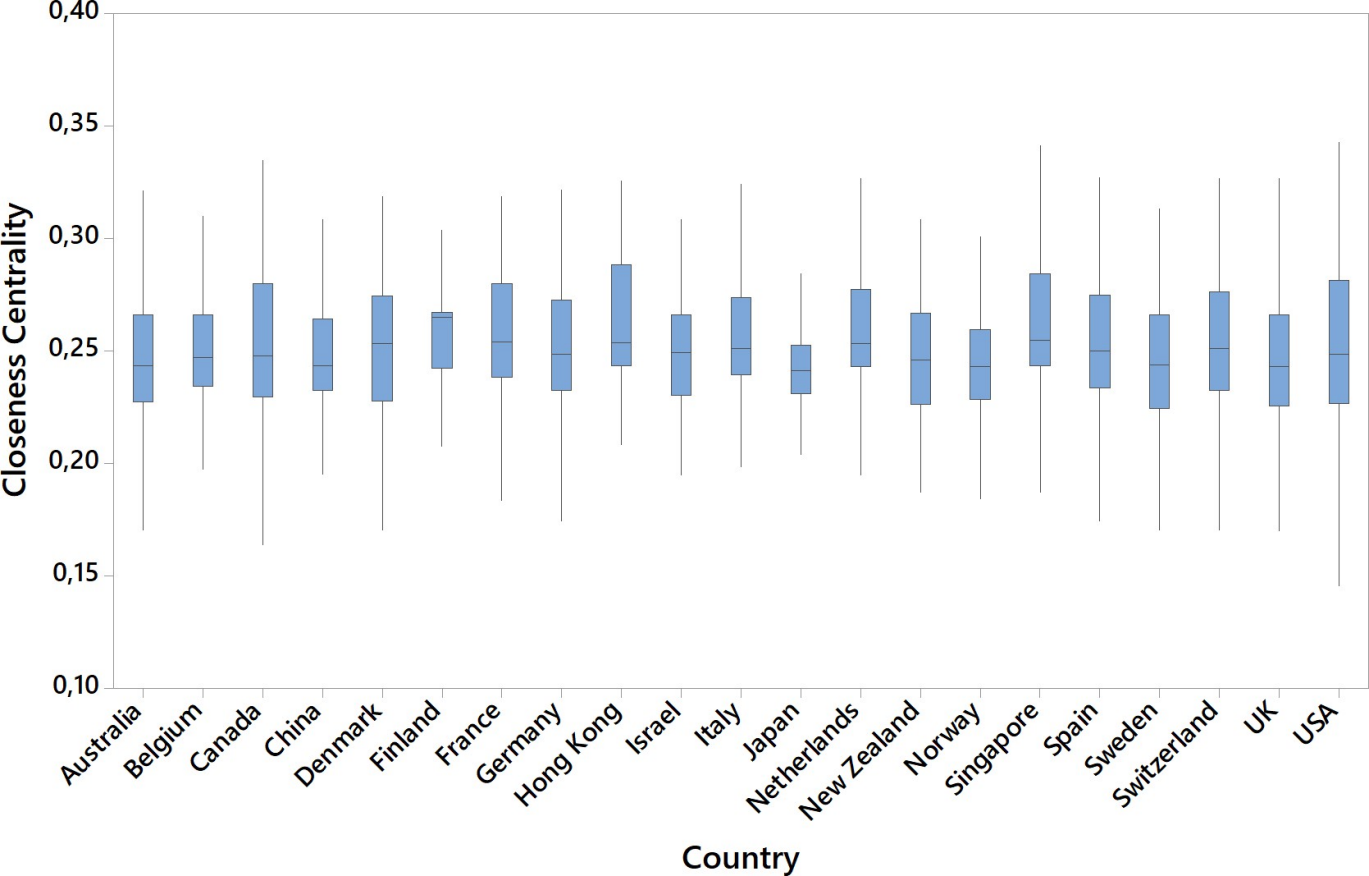
Boxplot of closeness centrality for countries with 100 or more EB members in the sample.

108 countries are represented in the sample. Table 6 presents the top ten countries in the overall network along with their number of Ebs and relative position in each of the six fields. USA represents 44.92% of the sample followed by UK (12.95%) and Australia (5.00%). The top ten countries have 200 or more EB members, accounting for 80% of the sample. Fifty-five countries have fewer than ten EB members, representing 1.03% of the sample (156 EB members). Twenty-three countries have only one EB member. We compared the countries with more than 100 members in the overall network (top 21 countries, 13,625 EB members, representing 90.33% of the sample), and found that there were no statistical differences for degree (H = 16.64, p = 0.676) or betweenness centrality (H = 15.53, p = 0.745). The results of the Kruskal-Wallis test for closeness centrality suggests that there is a statistical difference between countries (H = 208.77, p<0.01). Fig 5 shows differences graphically. They are small (range: Mdn_Japan_ = 0.241–Mdn_Finland_ = 0.265) and may be caused by the differences in the sample sizes between countries, although only instances with more than 100 EB members are considered.

**Table 6:** Geographic representation of EBs for each field and for the overall network.

	Comm.	Psych.	Pol. Sc.	Soc.	Eco.	Man.	All
USA	754 (1)	622 (1)	651 (1)	695 (1)	1534 (1)	2612 (1)	6775 (1)
UK	143 (2)	68 (3)	323 (2)	246 (2)	518 (2)	678 (2)	1953 (2)
Australia	120 (3)	72 (2)	72 (4)	80(3)	177 (3)	248 (3)	754 (3)
Canada	39 (6)	41 (4)	61 (5)	64 (4)	125 (6)	227 (4)	547(4)
Germany	53 (4)	21 (5)	82 (3)	43 (5)	130 (4)	170 (6)	493 (5)
France	12 (21)	6 (8)	40(6)	39 (6)	109 (7)	177 (5)	381 (6)
Netherlands	46 (5)	12 (7)	31 (8)	19 (10)	113 (8)	133 (7)	348 (7)
Italy	11 (22)	19 (6)	25 (9)	20 (9)	129 (5)	119 (9)	322 (8)
China	14 (18)	5 (11)	3 (33)	5 (23)	106 (9)	130 (8)	261 (9)
Spain	24 (10)	6 (10)	34 (7)	12 (15)	62 (14)	95 (10)	232 (10)

*Notes*: Each cell presents the number of EB members in the sample and the relative position in the field (in parenthesis).

### Institutional representation

**Fig 6:**
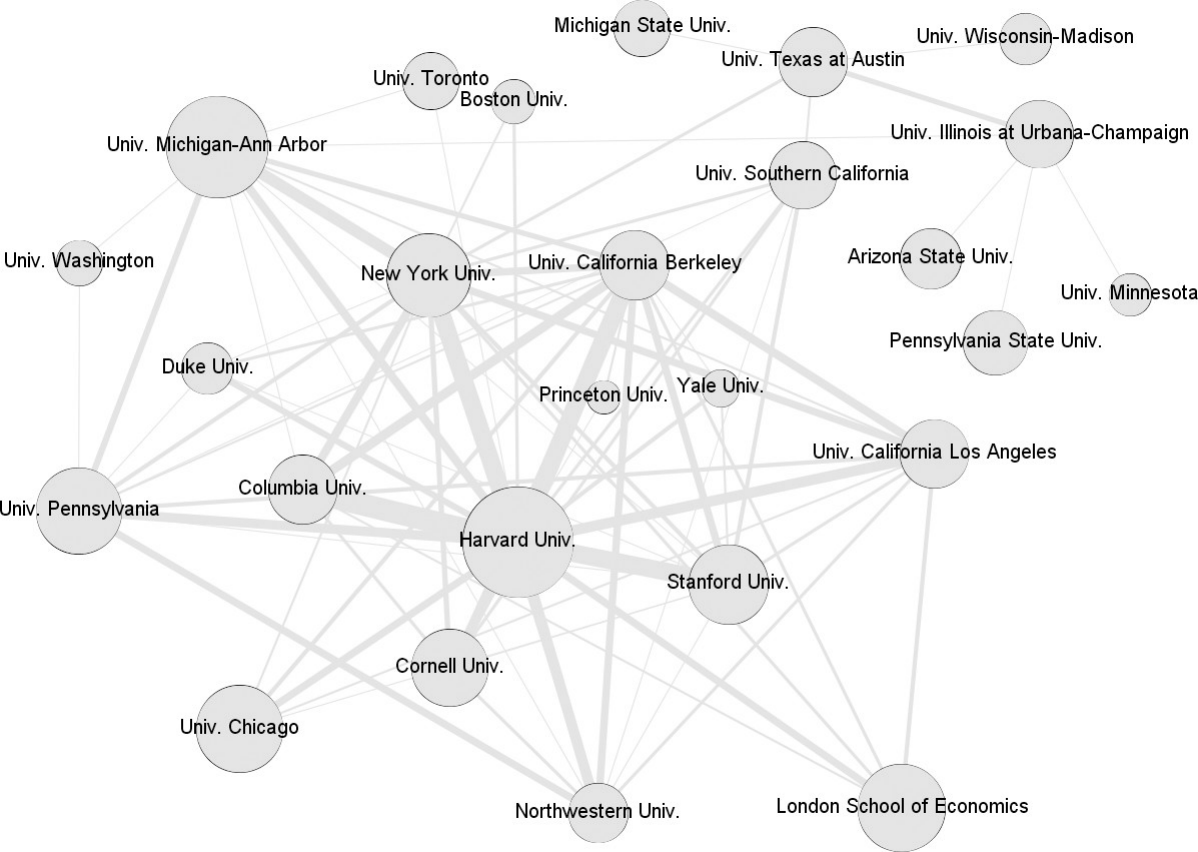
Projection of institutions for the overall network. Size of each node is proportional to the number of different scholars from the institution who are on EBs. Thickness of the lines is proportional to the number of common scholars in EBs between the institutions connected.

The EB members in the sample represent 2850 different institutions. Table 7 presents the top institutions in each field and for the complete network. 1681 institutions are represented by only one EB member. We can see differences in the predominant institutions for each field. Four fields include Harvard and Michigan-Ann Arbor in their top ten. Pennsylvania and the London School of Economics are in the top positions of three fields. In the overall network projection for all fields (Fig 6), Harvard University is in the central position in terms of the quantity and intensity of connections as measured by the number of scholars shared with other institutions in Ebs. Harvard participates in the top four strongest connections, which are with Columbia, New York, California-Berkeley and Stanford. These institutions can also be considered the next most relevant actors in the map of institutions.

**Table 7:** Institutional representation of EBs for each field and for the overall network.

Pos	Comm.	#	Psych.	#	Pol. Sc.	#	Soc.	#	Eco.	#	Man.	#	All	#
1	Texas-Austin	27	Harvard	16	LSE	30	Chicago	44	LSE	54	MIT	67	Harvard	145
2	Michigan State	25	California-San Diego	13	Harvard	25	Michigan-Ann Arbor	29	Stanford	50	Cornell	50	Michigan-Ann Arbor	126
3	Amsterdam	24	Columbia	10	Oxford	22	California-L. Angeles	21	Chicago	44	Arizona State	46	LSE	109
4	California-Sa. Barbara	19	Illinois-Urbana Champaign	10	Columbia	19	New York	18	Harvard	41	INSEAD	43	Chicago	108
5	Southern California	19	Stanford	9	King’s College	18	California-Berkeley	17	California-Berkeley	41	CBS	42	Pennsylvania	107
6	Temple	17	California-L. Angeles	9	Australian National	17	Toronto	16	Glasgow	41	Pennsylvania	42	MIT	104
7	Penn State	16	Sydney	9	Edinburgh	15	Cardiff	14	Columbia	32	Harvard	41	New York	104
8	Illinois-Urbana Champaign	16	Arizona State	8	Notre Dame	15	Harvard	14	Pennsylvania	32	Michigan-Ann Arbor	39	Stanford	99
9	Michigan-Ann Arbor	15	Emory	8	Johns Hopkins	14	LSE	12	Melbourne	30	Penn State	38	Cornell	96
10	Pennsylvania	15	Ohio State	8	Michigan-Ann Arbor	14	Manchester	12	New York	28	Michigan State	37	California-Berkeley	87

*Notes*: # represents the number of editorial board members from the institution

**Fig 7:**
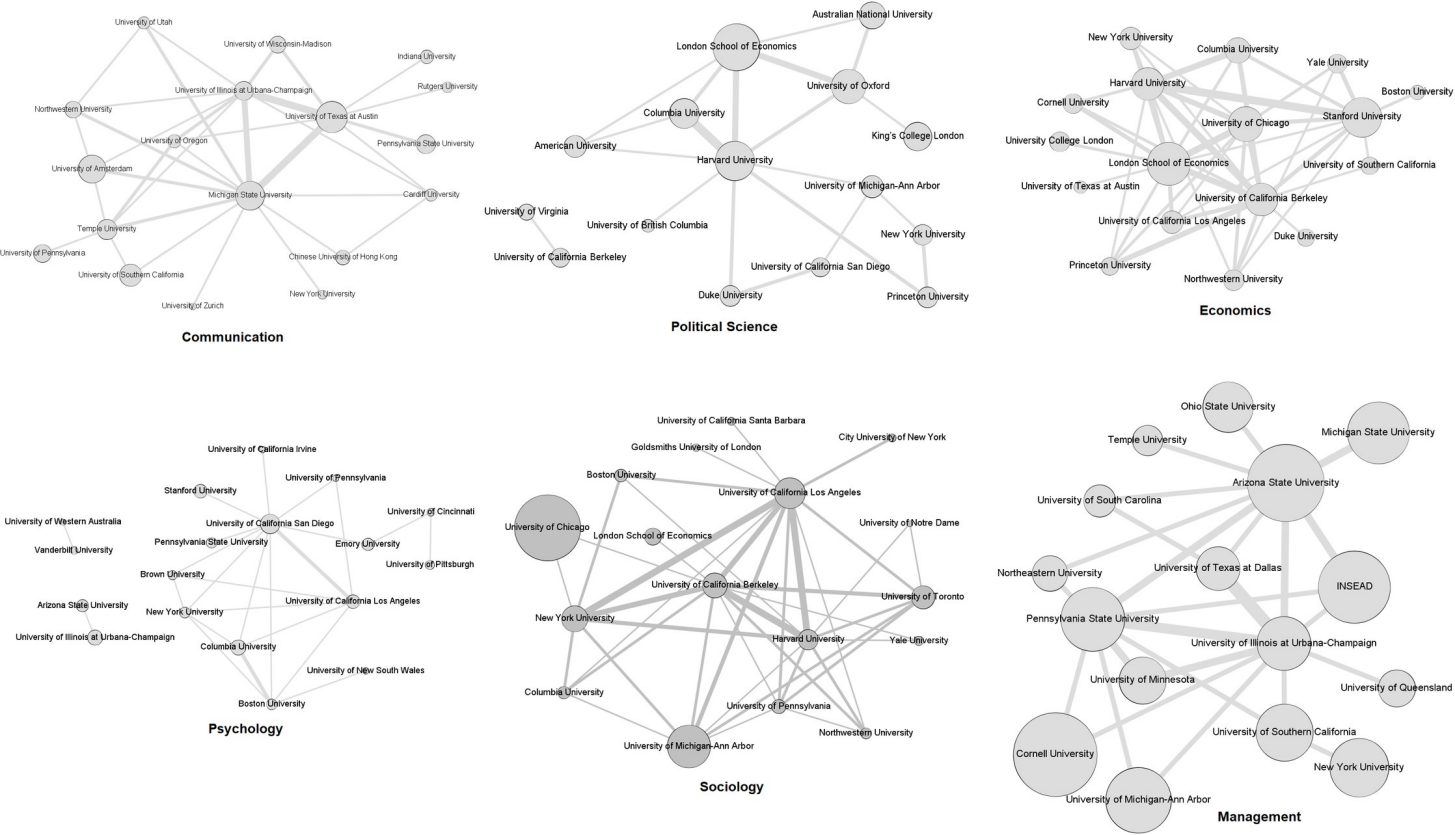
Projection of institutions for each field. Size of each node is proportional to the number of different scholars from the institution in EBs. Thickness of the lines is proportional to the number of common scholars in EBs between the institutions connected.

Fig 7 shows the institutions for each field. The size of the slice for each network was set to the minimum number that resulted in a projection with less than 20 institutions. In this way, we can compare fields by observing the prominent institutions and their connections. Most of the central institutions in each projection are also those with the top positions regarding the number of scholars for all fields. The opposite is not true in several fields, however. In sociology, the University of Chicago has more scholars than all the others, but in the projection, it occupies a position on the periphery. MIT and Harvard are not even present in the projections for management and psychology, respectively, although they present the largest number of scholars.

The most prominent institutions for each field in terms of the intensity of the connections of the slice as measured by the number of scholars shared through EBI are as follows: communication, Michigan State, Texas at Austin and Illinois at Urbana-Champaign; psychology, California-San Diego, California-Los Angeles, Columbia and Boston; political science, Columbia, Harvard, Oxford and London School of Economics; sociology, California-Berkeley, California-Los Angeles, New York and Harvard; and economics and management, the projection of institutions (Fig 7) shows that more institutions (more than four) present strong connections when compared to other fields. We then have more diversity at the core of institutional representation in these two fields. The density of each field projection varies substantially, with economics (0.309) and sociology (0.294) reflecting more interaction among central institutions. Communication (0.216), management (0.208), and political science (0.190) present around 20% of all possible connections between the top institutions in their slices. Psychology presents the less connected core with a density of 0.157 in its slice.

We observe strong significant correlations between the number of scholars in the dataset and network metrics: degree (r = 0.970, p<0.01), closeness centrality (r = 0.643, p<0.01) and betweenness centrality (r = 0.848, p<0.01). Metrics were computed for the projection of institutions in the overall network of all fields. The results are similar when repeating the test only with institutions that have 20 or more members in the dataset (N = 176, r_degree_ = 0.924, r_closeness_ = 0.533, r_betweeness_ = 0.701, p<0.01 in all cases), showing that correlations for the whole dataset are not outweighed by institutions with a small number of scholars who present low values for network metrics. The total number of scholars across all EBs reflects the prominence of the institution, and can be used as an estimator of its influence. Although this result may seem obvious, it may very well be the case that overrepresented institutions only manage to place their scholars in a limited number of peripheral journals that do not have scholars from other prominent institutions in their EBs. Similar results are observed for each individual field, with strong significant correlations in most cases (Table 8). The only exception is psychology which presents only moderate correlations for degree (r = 0.476, p<0.01), and a weak correlation for closeness centrality (r = 0.262, p<0.01).

**Table 8:** Correlation between the number of scholars from each institution and the network metrics of the projection of institutions for each field and for the overall network.

Metric	Comm.	Psych.	Pol. Sc.	Soc.	Eco.	Man.	All
Degree	0.892	0.476	0.920	0.751	0.945	0.900	0.970
Closeness Centrality	0.809	0.262	0.738	0.577	0.599	0.725	0.643
Betweeness Centrality	0.839	0.659	0.824	0.666	0.755	0.656	0.848

*Notes*: Network metrics were computed for the projection of all institutions and separately for the network of each field and for the overall network. Each cell contains the Pearson correlation coefficient (r). The p-value is <0.01 in all cases.

## Discussion and conclusions

A growing body of literature acknowledges the role of editorial boards in knowledge production and dissemination [3, 4]. However, studies of editorial board representation have mainly provided within-field analysis [13], mainly focusing on a single dimension of such representation, such as gender [32] or geographic diversity [2]. As the analysis of EBI is typically restricted to individual disciplines [25], they have failed to consider the relevance of potential interlocking between related, but different academic fields. This study addresses this knowledge gap and offers a complex cross-disciplinary approach to the geographical, gender and institutional composition of editorial boards, with a specific emphasis on within- and between-fields editorial board interlocking.

In line with the results of former studies on the composition of journal authorship, editorial boards, and reviewers [54, 55], we found clear evidence for the uneven distribution of editorial board membership: the typical central editorial board member is a male scholar from an elite American university who participates in many different journals. There are significant disciplinary differences, however, requiring a more nuanced interpretation of the experienced inequalities.

### The gender composition of journal EBs

The vast majority of available studies found a significant male majority on editorial boards [e.g., 32]. As we lack gender breakdowns for the fields under study, normative considerations of editorial board composition may be biased. However, our study does provide insightful findings that may resonate in other dimensions of scientific production. First, our results indicate that women are less likely to be editorial board members across all the analyzed disciplines, with the highest gender imbalance in economics and management. This might suggest that disciplines traditionally related to business are still more dominated by males than disciplines with an emphasis on social impact [30]. The results might also suggest gender role stereotyping [56], as male dominance is more prevalent in traditionally more masculine fields [31]. Another possible explanation of these findings is that there is a more significant male EB share in disciplines that are closer to decision making and power than in fields focusing on social care [31], potentially resembling the gender structure of such fields.

Although female scholars are less prevalent in editorial boards, however, there are no significant differences in terms of positioning in the network. Female scholars are even found in more central positions than male scholars in psychology, sociology, and management. This demonstrates that while the price of entry is high for female scholars, as their productivity might be burdened by a vicious circle of gender inequalities [55], once they gain admission to the “elite club” [1] of editorial boards, their network positions are comparable to those of their male peers. In other words, the gender imbalance is significant at the entrance level, but the possible effect of gender is limited when female scholars become editorial board members. This is not the case with economics, however, where both the proportion of female EB members and their network positions on editorial boards are significantly lower than those of their male counterparts. Together with the disciplinary differences in gender imbalance, this might suggest a connection with possible gender inequality in economics, favoring male scholars.

### The geographical distribution of EB positions

The striking overrepresentation of Western, and especially American scholarship, in authorship and editorial boards has been noted by several studies, and our main findings are consistent with these results. Specifically, we found that native English-speaking countries were dominant, as the countries most represented on editorial boards are the US, the UK, Australia, and Canada, the four largest English-speaking geographies. This order is almost consistent across disciplines, except that Germany is more represented than Canada in communication, political science, and economics. Native English countries have a linguistic advantage as papers in most international journals are written in English. This phenomenon may create a deleterious effect, in which less productivity as a results of language inequalities may lead to less chances to be invited onto the editorial boards of international journals.

Another important finding is that China is represented amongst the top 10 countries on editorial boards in general, and its presence is especially significant in the two disciplines that are most closely related to business: management and economics. As the sole non-Western country with visible representation on the editorial boards of international journals, China’s presence is different from their Western counterparts: while Western countries have an even representation across disciplines, China is only represented in a small set of disciplines, mainly political science and sociology. A potential explanation could be that Chinese scholars are less likely to be invited to editorial boards in fields more closely related to politics and social order, than more business-like disciplines where policies are more global and market interests might supersede potential ideological differences. Even if China’s production in social sciences has been growing in the last two decades, this development is significantly slower than in the natural sciences. As Ping Zhou observes, “although Chinese publications began to grow visibly in 1999, the gap between China and the West represented by the USA and the EU is too wide to be reduced within a short period” [57]. This trend may also be mirrored in editorial board representation.

### Institutional composition

The representation of different higher education institutions follows a Pareto distribution, as most editorial board members are from a limited set of elite institutions and more than half of the universities in our sample are represented by only one EB member. We support the findings of other studies [26], by finding a clear US overrepresentation, as with the sole exception of the London School of Economics (LSE), the top 10 institutions providing most EB members only includes US universities. Moreover, the top 10 universities in communication, psychology and management are all from the US.

The number of editorial board members correlates with the network position of a given university; thus Harvard, which awards the most EB members, also occupies the central position in the EBI network. US universities, especially Harvard, Columbia, Cornell, Stanford, UC Berkeley and New York University (all of which are private universities except Berkeley) form a well-connected network, while despite having many EB members, the LSE is relatively distanced from the hub of elite US universities.

In line with the corresponding literature [27] we found a stratified US dominance, meaning that it is not just that EBs from US universities are significantly overrepresented in both the overall network and in different disciplines, but that elite institutions–six of the eight Ivy League universities–provide the most EB members and form the strongest ties with each other. This implies that the “top of the tops” might determine, or at least have a very significant effect on, who and what can be published in international journals [7].

As discussed in the literature of social identity theory [58], scholars from the same academic backgrounds and perspectives might prefer each other’s norms, values and approaches [1, 59], and, consequently, might decide in favor of authors from the same institutions. As significantly publishing in specific journals might be a prerequisite for joining their EBs [6], favoring authors from the same institution might also lead to favoring them as editorial board members. The “prestige bias” [28] in favor of elite institutions thus holds for both the level of authors and EB members, and in line with the aforementioned preferences, author level prestige bias and EB selection criteria even may strengthen each other’s effects.

### Editorial board interlocking

The density values we observed were low for all disciplines and were similar to the values reported for the same and other fields: communication [7], economics [8], finance [21], library science [45] and knowledge management [10]. Our results show that most journals have characteristic editorial boards, while, on a different scale, both within-field and between-field interlocking is significant. The most obvious finding to emerge from the analysis is that, apart from psychology, five of the six disciplines are considerably interconnected with each other.

Basically, there are several types of interlocking. The first is represented by communication, where both within field and between field interlocking is significant, and there are more central journals surrounded by a set of more minor outlets. In the case of communication, there are several journals with high betweenness centrality, such as *Management Communication Quarterly*, the *International Journal of Advertising* and the *International Journal of Conflict Management*, which strongly connects the field of communication to the field of management with significant EBI. This latter field has a similar structure as that of communication, as it also has some more central journals but lacks journals with strong betweenness centrality positions.

Another type is represented by economics and political science, where we did not find a clear differentiation between central and peripheral journals, but where there are journals in strong betweenness centrality positions. There are journals that connect economics to management, such as *Industry and Innovation*, and journals that connects economics to political science, such as the *Review of International Political Economy*. Half a dozen political science journals share a significant number of EB members with economics.

Finally, sociology forms a dense network with journals that lacks within field interlocking, but shows substantially more between field interlocking, and with journals with strong ties to communication, economics and political science. While these disciplines are all interdisciplinary in terms of EBI, communication typically interlocks with management and sociology, economics has strong ties to management and political science, and sociology is the most balanced in the sense of interdisciplinarity as it interlocks almost equally with the other four disciplines.

One unanticipated finding was that psychology is significantly detached from the other five disciplines, with a very limited number of shared EB members. This finding may be explained by several factors. First, it might demonstrate a thematic, theoretical, and methodological divergence between mainstream psychology and the other five disciplines. While the study did not content-analyze journal articles, it is reasonable to suggest that psychology is somehow distanced from more social scientific fields, and that psychologists might use more field-specific methods and theories, meaning that the editorial board composition of psychology journals may be detached from the other analyzed fields.

Based on its relative distance from the other analyzed disciplines, we might even assume that contemporary psychology, as represented by the editorial boards of journals, is more closely related to the natural and life sciences than to social scientific disciplines. Second, as reviewers are, in most cases, selected on the basis of their research production and impact [6, 21], our results suggest that it is not usual for psychologists to publish in other disciplines, and consequently they are not highly visible in the publication networks of different fields. While this study does not focus on author networks, our results might indicate that psychology is relatively closed in terms of not just editorial boards but also authorship.

### Limitations

Although the study has successfully demonstrated a significant gender and geographical imbalance across the analyzed fields and observed a considerable EBI both within and between fields, it has some limitations that should be addressed in future research. First, we selected a particular number of fields within social sciences. Future research may consider other fields to map different disciplinary interlocks. Second, we implemented a representative, proportional random sample to each field. However, covering the full set of journals might provide a more detailed picture of EBI. The amount of data and the need for manual coding meant that a full mapping of all journals in all fields was not feasible, and future research might try to analyze all journals with fewer disciplines. Further, the sampling method presents its own limitations in terms of the representativity of communities and the distribution of attributes. Although this comes from the need to accommodate what is feasible when gathering data, we have to be careful with results and interpretations concerning communities, and gender, geographic and institutional representation. Future research can then focus on obtaining complete networks of EBs with the help computational technologies such as web scraping or selecting all journals within a quartile of the JCR ranking. Third, while working with the JCR journal list is reasonable and well established in the literature, findings may be different should the study focus on other academic rankings. Future research may thus replicate our comparative analysis with a different database, such as Scopus. Fourth, according to recent literature [60], scientific journals sometimes misreport member information in their website or present outdated affiliation information. We corrected such inappropriate errors when were detected.

## Supporting information

S1 Appendix Journals included in the study. (DOCX)

S1 Fig Labeled graph of the social network of journals and EBs. The labeled graph of the social network of journals and members is provided as high-resolution image (png) and vector image (svg).
(ZIP)

## References

[1] Burgess TF, Shaw NE. Editorial board membership of management and business journals: A social network analysis study of the Financial Times 40. British journal of management. 2010 Sep;21(3):627–48.

[2] Goyanes M. Editorial boards in communication sciences journals: Plurality or standardization?. International communication gazette. 2020 Jun;82(4):342–64.

[3] Pan Y, Zhang JQ. The composition of the editorial boards of general marketing journals. Journal of Marketing Education. 2014 Apr;36(1):33–44.

[4] Metz I, Harzing AW, Zyphur MJ. Of journal editors and editorial boards: who are the trailblazers in increasing editorial board gender equality?. British journal of management. 2016 Oct;27(4):712–26.

[5] Dhanani A, Jones MJ. Editorial boards of accounting journals: gender diversity and internationalisation. Accounting, auditing & accountability journal. 2017 Jun 19.

[6] Raelin JA. Refereeing the game of peer review. Academy of Management Learning & Education. 2008 Mar;7(1):124–9.

[7] Goyanes M, De-Marcos L. Academic influence and invisible colleges through editorial board interlocking in communication sciences: a social network analysis of leading journals. Scientometrics. 2020 May;123(2):791–811.

[8] Baccini A, Barabesi L. Interlocking editorship. A network analysis of the links between economic journals. Scientometrics. 2010 Feb 1;82(2):365–89.

[9] Youk S, Park HS. Where and what do they publish? Editors’ and editorial board members’ affiliated institutions and the citation counts of their endogenous publications in the field of communication. Scientometrics. 2019 Sep;120(3):1237–60.

[10] Teixeira EK, Oliveira M. Editorial board interlocking in knowledge management and intellectual capital research field. Scientometrics. 2018 Dec;117(3):1853–69.

[11] de Albuquerque A, de Oliveira TM, dos Santos Junior MA, de Albuquerque SO. Structural limits to the de-westernization of the communication field: The editorial board in Clarivate’s JCR system. Communication, Culture and Critique. 2020 Jun 1;13(2):185–203.

[12] Goyanes M, Demeter M. How the geographic diversity of editorial boards affects what is published in JCR-ranked communication journals. Journalism & mass communication quarterly. 2020 Dec;97(4):1123–48.

[13] Lauf E. National diversity of major international journals in the field of communication. Journal of communication. 2005 Mar;55(1):139–51.

[14] Greenbaum HK, Goodsir HL, Smith MC, Robinson DH. Female participation as top-producing authors, editors, and editorial board members in educational psychology journals from 2009 to 2016. Educational Psychology Review. 2018 Dec;30(4):1283–9.

[15] Fong CJ, Yoo JH, Jones SJ, Torres LG, Decker ML. Trends in female authorships, editorial board memberships, and editorships in educational psychology journals from 2003 to 2008. Educational Psychology Review. 2009 Sep;21(3):267–77.

[16] Pardeck JT, Arndt BJ, Light DB, Mosley GF. Distinction and achievement levels of editorial board members of psychology and social work journals. Psychological Reports. 1991 Apr.

[17] Palmer B, van Assendelft L, Stegmaier M. Revisiting the Presence of Women in Political Science Journal Editorial Positions. PS: Political Science & Politics. 2020 Jul;53(3):499–504.

[18] Stegmaier M, Palmer B, Van Assendelft L. Getting on the board: the presence of women in political science journal editorial positions. PS: Political science & politics. 2011 Oct;44(4):799–804.

[19] Dart J. Sports sociology, journals and their editors. World Leisure Journal. 2013 Mar 1;55(1):6–23.

[20] Willis CL, McNamee SJ. Social networks of science and patterns of publication in leading sociology journals, 1960 to 1985. Knowledge. 1990 Jun;11(4):363–81.

[21] Andrikopoulos A, Economou L. Editorial board interlocks in financial economics. International review of financial analysis. 2015 Jan 1;37:51–62.

[22] Baccini A. Italian economic journals. A network-based ranking and an exploratory analysis of their influence on setting international professional standards. Rivista italiana degli economisti. 2009;14(3):491–512.

[23] Brown C. Communication in the sciences. Annual Review of Information Science and Technology. 2010 Jan 1;44(1):285–316.

[24] Altman M, Cohen PN. Openness and diversity in journal editorial boards. 2021.

[25] Willett P. The characteristics of journal editorial boards in library and information science. International journal of knowledge content development & technology. 2013;3(1):5–17.

[26] Lo WY. Soft power, university rankings and knowledge production: Distinctions between hegemony and self‐determination in higher education. Comparative Education. 2011 May 1;47(2):209–22.

[27] Kong L, Qian J. Knowledge circulation in urban geography/urban studies, 1990–2010: Testing the discourse of Anglo-American hegemony through publication and citation patterns. Urban Studies. 2019 Jan;56(1):44–80.

[28] Clauset A, Arbesman S, Larremore DB. Systematic inequality and hierarchy in faculty hiring networks. Science advances. 2015 Feb 12;1(1):e1400005. doi: 10.1126/sciadv.1400005 26601125 PMC4644075

[29] Tollefson J. China declared world’s largest producer of scientific articles. Nature. 2018 Jan 1;553(7686):390–1. 10.1038/d41586-018-00927-4 32094808

[30] Holman L, Stuart-Fox D, Hauser CE. The gender gap in science: How long until women are equally represented?. PLoS biology. 2018 Apr 19;16(4):e2004956. doi: 10.1371/journal.pbio.2004956 29672508 PMC5908072

[31] Larivière V, Ni C, Gingras Y, Cronin B, Sugimoto CR. Bibliometrics: Global gender disparities in science. Nature. 2013 Dec;504(7479):211–3. doi: 10.1038/504211a 24350369

[32] Topaz CM, Sen S. Gender representation on journal editorial boards in the mathematical sciences. PLoS One. 2016 Aug 18;11(8):e0161357. doi: 10.1371/journal.pone.0161357 27536970 PMC4990198

[33] Besancenot D, Huynh KV, Faria JR. Search and research: The influence of editorial boards on journals’ quality. Theory and Decision. 2012 Oct;73(4):687–702.

[34] Hogg MA. A social identity theory of leadership. Personality and social psychology review. 2001 Aug;5(3):184–200.

[35] Zuccala A. Modeling the invisible college. Journal of the American Society for information Science and Technology. 2006 Jan 15;57(2):152–68.

[36] Baccini A, Barabesi L, Khelfaoui M, Gingras Y. Intellectual and social similarity among scholarly journals: An exploratory comparison of the networks of editors, authors and co-citations. Quantitative Science Studies. 2020 Feb 1;1(1):277–89.

[37] Baccini F, Barabesi L, Baccini A, Khelfaoui M, Gingras Y. Similarity network fusion for scholarly journals. Journal of Informetrics. 2022 Feb 1;16(1):101226.

[38] Leydesdorff L, Rafols I. A global map of science based on the ISI subject categories. Journal of the American Society for Information Science and Technology. 2009 Feb;60(2):348–62.

[39] Pudovkin AI, Garfield E. Algorithmic procedure for finding semantically related journals. Journal of the American Society for Information Science and Technology. 2002 Nov;53(13):1113–9.

[40] Maiya AS, Berger-Wolf TY. Sampling community structure. InProceedings of the 19th international conference on World wide web 2010 Apr 26 (pp. 701–710).

[41] Leskovec J, Faloutsos C. Sampling from large graphs. InProceedings of the 12th ACM SIGKDD international conference on Knowledge discovery and data mining 2006 Aug 20 (pp. 631–636).

[42] Gjoka M, Kurant M, Butts CT, Markopoulou A. Practical recommendations on crawling online social networks. IEEE Journal on Selected Areas in Communications. 2011 Sep 26;29(9):1872–92.

[43] Maiya AS, Berger-Wolf TY. Benefits of bias: Towards better characterization of network sampling. InProceedings of the 17th ACM SIGKDD international conference on Knowledge discovery and data mining 2011 Aug 21 (pp. 105–113).

[44] Wagner C, Singer P, Karimi F, Pfeffer J, Strohmaier M. Sampling from social networks with attributes. InProceedings of the 26th international conference on world wide web 2017 Apr 3 (pp. 1181–1190).

[45] Baccini A, Barabesi L. Seats at the table: The network of the editorial boards in information and library science. Journal of informetrics. 2011 Jul 1;5(3):382–91.

[46] Liwei Z, Chunlin J. Social network analysis and academic performance of the editorial board members for journals of library and information science. COLLNET Journal of Scientometrics and Information Management. 2015 Jul 3;9(2):131–43.

[47] Scott J, Carrington PJ. The SAGE handbook of social network analysis. SAGE publications; 2011 May 25.

[48] Wasserman S, Faust K. Social network analysis: Methods and applications.

[49] Bastian M, Heymann S, Jacomy M. Gephi: an open source software for exploring and manipulating networks. InProceedings of the international AAAI conference on web and social media 2009 Mar 19 (Vol. 3, No. 1, pp. 361–362).

[50] Chakrabarti D, Faloutsos C. Graph mining: Laws, generators, and algorithms. ACM computing surveys (CSUR). 2006 Jun 29;38(1):2-es.

[51] Mislove A, Marcon M, Gummadi KP, Druschel P, Bhattacharjee B. Measurement and analysis of online social networks. InProceedings of the 7th ACM SIGCOMM conference on Internet measurement 2007 Oct 24 (pp. 29–42).

[52] Nettleton DF. Data mining of social networks represented as graphs. Computer Science Review. 2013 Feb 1;7:1–34.

[53] Robins G, Pattison P, Woolcock J. Small and other worlds: Global network structures from local processes. American Journal of Sociology. 2005 Jan;110(4):894–936.

[54] Goyanes M, Demeter M, Cheng Z, de Zúñiga HG. Measuring publication diversity among the most productive scholars: how research trajectories differ in communication, psychology, and political science. Scientometrics. 2022 May 21:1–22.

[55] Zhang L, Shang Y, Huang Y, Sivertsen G. Gender differences among active reviewers: an investigation based on Publons. Scientometrics. 2022 Jan;127(1):145–79.

[56] Knobloch-Westerwick S, Glynn CJ. The Matilda effect—Role congruity effects on scholarly communication: A citation analysis of Communication Research and Journal of Communication articles. Communication Research. 2013 Feb;40(1):3–26.

[57] Zhou P, Thijs B, Glänzel W. Is China also becoming a giant in social sciences?. Scientometrics. 2009 Jun 1;79(3):593–621.

[58] Tajfel H, Turner JC. The social identity theory of intergroup behavior. InPolitical psychology 2004 Jan 9 (pp. 276–293). Psychology Press.

[59] Harzing AW, Metz I. Practicing what we preach. Management International Review. 2013 Apr;53(2):169–87.

[60] Csomós G, Lengyel B. Geographies of the global co-editor network in oncology. PloS one. 2022 Mar 17;17(3):e0265652. doi: 10.1371/journal.pone.0265652 35298566 PMC8929652

