# Supplementary material for: Editorial board interlocking across the social sciences: Modelling the geographic, gender, and institutional representation within and between six academic fields
Source: PLoS One. 2022 Sep 2;17(9):e0273552. doi: 10.1371/journal.pone.0273552 (PMC9439229; doi:10.1371/journal.pone.0273552)
Supplement: S1 Appendix — (DOCX) [file pone.0273552.s001.docx]

**Appendix: Journals included in the study**

*Academia-Revista Latinoamericana de Administracion, Academy of Management Journal, Action Research, Administrative Science Quarterly, American Economic Journal-Economic Policy, American J. Sociology, Amfiteatru Economic, Annals of Regional Science, Annual Review of Organizational Psychology and Organizational Behavior, Annual Review of Political Science, Anthrozoos, Applied Neuropsychology-Adult, Archives Europeennes de Sociologie, Archives of Clinical Neuropscychology, Asian-Pacific Economic Literature, Australian Economic History Review, Australian Economic Review, Australian J. Politics and History, Austrian J. Political Science, B E J. Economic Analysis & Policy, Biodemography and Social Biology, British J. Politics & Int. Relations, British J. Sociology, Bulletin of Economic Research, Cambridge Review of Int. Affairs, Canadian J. Agricultural Economics-Revue Canadienne d Agroeconomie, Canadian J. Sociology-Cahiers Canadiens de Sociologie, Career Development Int., Cepal Review, China Agricultural Economic Review, Chinese Management Studies, Climate Change Economics, Clinical Psychologist, Cliometrica, Cognitive Psychology, Communication & Sport, Communications-European J. Communication Research, Comparative Studies in Society and History, Contemporary Economic Policy, Contemporary Sociology-A J. Reviews, Continuum-J. Media & Cultural Studies, Contributions to Indian Sociology, Convergence-Int. J. Research New Media Technologies, Convergencia-Revista de Ciencias Sociales, Cornell Hospitality Quarterly, Cross Cultural & Strategic Management, Cultural Sociology, Culture and Organization, Cyberpsychology-J. Psychosocial Research on Cyberspace, Depression and Anxiety, Developmental Psychobiology, Digital Journalism, Discourse & Communication, Discourse Context & Media, Eastern European Countryside, Eastern European Economics, Econometric Reviews, Econometrics Journal, Economic and Social Review, Economic Development and Cultural Change, Economic History Review, Economic Modelling, Economic Record, Economics-The Open Access Open-Assessment E-Journal, Economist-Netherlands, Economy and Society, Education Finance and Policy, Electoral Studies, Electronic Commerce Research, Emerging Markets Finance and Trade, Energy Economics, Engineering Construction and Architectural Management, Environmental Communication-J. Nature and Culture, Ethnic and Racial Studies, European Economic Review, European J. Innovation Management, European J. Political Research, European Management Journal, European Management Review, European Political Science, European Security, Europe-Asia Studies, Experimental Aging Research, Frontiers in Human Neuroscience, Futures, Geopolitics, German J. Human Resource Management-Zeitschrift fur Personalforschung, Global Policy, Group & Organization Management, Health Sociology Review, Historia y Politica, Hitotsubashi J. Economics, Human Communication Research, Human Ecology, Human Factors, Human Resource Management Journal, Human Resource Management Review, Industrial Marketing Management, Industry and Innovation, Information & Management, Information and Organization, Information Communication & Society, Information Technology & Management, Innovation-Organization & Management, Innovation-The European J. Social Science Research, Insurance Mathematics & Economics, Int. Finance, Int. J. Advertising, Int. J. Communication, Int. J. Conflict and Violence, Int. J. Conflict Management, Int. J. Game Theory, Int. J. Health Economics and Management, Int. J. Islamic and Middle Eastern Finance and Management, Int. J. Logistics Management, Int. J. Management Reviews, Int. J. Project Management, Int. J. Technology Management, Int. Review of Economics Education, Int. Review of Law and Economics, Int. Small Business Journal-Researching Entrepreneurship, Int. Studies Review, Int. Theory, J. Australian Political Economy, J. Broadcasting & Electronic Media, J. Business and Technical Communication, J. Commodity Markets, J. Common Market Studies, J. Communication, J. Comparative Economics, J. Conflict Resolution, J. Consumer Affairs, J. Econometrics, J. Economic Behavior & Organization, J. Economic Dynamics & Control, J. Economic Geography, J. Economic Growth, J. Economic Interaction and Coordination, J. Economic Methodology, J. Economic Surveys, J. Economics & Management Strategy, J. Enterprise Information Management, J. European Integration, J. Evolutionary Economics, J. Financial Econometrics, J. Forecasting, J. Health Economics, J. Human Rights, J. Information Technology, J. Int. Economics, J. Int. Management, J. Leadership & Organizational Studies, J. Management Inquiry, J. Management Studies, J. Media Ethics, J. Monetary Economics, J. Money Credit and Banking, J. Motor Behavior, J. Organizational and End User Computing, J. Organizational Behavior Management, J. Pension Economics & Finance, J. Policy History, J. Political Economy, J. Product and Brand Management, J. Purchasing and Supply Management, J. Real Estate Finance and Economics, J. Small Business Management, J. Sport & Social Issues, J. Sports Economics, J. Strategic Studies, J. Studies on Alcohol and Drugs, J. Supply Chain Management, J. the Association of Environmental and Resource Economists, J. the Japanese and Int. Economies, J. Transport Economics and Policy, J. Wine Economics, J. World Trade, Jahrbucher für Nationalokonomie und Statistik, Japan and the World Economy, Japanese Economic Review, Javnost-The Public, Journalism Studies, Kolner Zeitschrift für Soziologie und Sozialpsychologie, Kyklos, Labour Economics, Latin American Politics and Society, Leadership & Organization Development Journal, Legislative Studies Quarterly, Lex Localis-J. Local Self-Government, Management Communication Quarterly, Management Int. Review, Management Science, Manufacturing & Service Operations Management, Mathematical Finance, Media Culture & Society, Mediterranean Politics, Metroeconomica, MIT Sloan Management Review, Negotiation Journal, Neurocase, Neuropsychological Rehabilitation, Neuropsychology, Nonprofit Management & Leadership, North American J. Economics and Finance, Oeconomia Copernicana, Organization Science, Organizational Behavior and Human Decision Processes, Organizational Dynamics, Organizational Research Methods, Papers in Regional Science, Politica y Gobierno, Political Analysis, Political Behavior, Political Communication, Political Science Quarterly, Political Studies, Political Theory, Politicka Ekonomie, Politics & Society, Politics and Governance, Politics and Religion, Politics Philosophy & Economics, Politics Religion & Ideology, Polititcal Quarterly, Politix, Prague Economic Papers, Psychological Review, Psychosomatics, Psychotherapy and Psychosomatics, Qualitative Research, Quantitative Economics, Quantitative Marketing and Economics, Rationality and Society, Regional Studies, Regulation & Governance, Research Quarterly for Exercise and Sport, Review of African Political Economy, Review of Development Economics, Review of Environmental Economics and Policy, Review of Finance, Review of Int. Political Economy, Review of Religious Research, Revista de Economia Aplicada, Revista de Estudios Politicos, Revue Francaise de Sociologie, Romanian J. Economic Forecasting, Scandinavian J. Hospitality and Tourism, Science and Public Policy, Scottish J. Political Economy, Service Science, Sexualities, Singapore Economic Review, Social Indicators Research, Social Media + Society, Society and Mental Health, Sociologia, Sociological Methods & Research, Sociological Spectrum, Sociological Theory, Sociologisk Forskning, Sociology Compass, Sociology of Religion, Sociology-The J. the British Sociological Association, South African J. Economic and Management Sciences, Sport in Society, Strategic Management Journal, Structural Change and Economic Dynamics, Studies in American Political Development, Studies in Conflict & Terrorism, Swiss Political Science Review, Technology Analysis & Strategic Management, Territory Politics Governance, Text & Talk, Transport Policy, Transportation Research Part E-Logistics and Transportation Review, Value in Health, Vision Research, World Bank Economic Review, World Bank Research Observer, Youth & Society, Zeitschrift für Psychosomatische Medizin und Psychotherapie.*
